# Neglected tropical diseases: exploring long term practical approaches to achieve sustainable disease elimination and beyond

**DOI:** 10.1186/s40249-017-0361-8

**Published:** 2017-09-27

**Authors:** Giuseppina Ortu, Oliver Williams

**Affiliations:** Malaria Consortium, Development House, 56-54 Leonard street, London, EC24 4LT UK

**Keywords:** NTDs, Primary health care, Integration, Surveillance, Community engagement

## Abstract

**Background:**

Remarkable progress has been made in the fight against neglected tropical diseases, but new challenges have emerged. Innovative diagnostics, better drugs and new insecticides are often identified as the priority; however, access to these new tools may not be sufficient to achieve and sustain disease elimination, if certain challenges and priorities are not considered.

**Main body:**

The authors summarise key operational challenges, and based on these, identify two major priorities: strengthening the capacity of the primary health care health system in correctly diagnosing and managing neglected tropical diseases; and establishing an effective disease surveillance process.

Five steps are proposed as concrete actions to build an effective primary health care service for neglected tropical diseases, and a health management information system capable of accurately reporting these diseases. Community engagement and formalization of community health workers role are proposed as essential components of these steps.

Shift of financial support from disease oriented programmes to disease integrated interventions, improved access to international guidelines for primary health care staff, and availability of donated drugs in health care structures are also suggested as key elements of the proposed process.

**Conclusion:**

The authors conclude that failure to address these priorities now may lead to further challenges on the long path towards neglected tropical disease elimination and beyond.

**Electronic supplementary material:**

The online version of this article (doi: 10.1186/s40249-017-0361-8) contains supplementary material, which is available to authorized users.

## Multilingual abstracts

Please see Additional file 1 for translations of the abstract into the five official working languages of the United Nations.

## Background

### NTD landscape

Over the last few years there has been an increased interest in tackling neglected tropical diseases (NTDs), as their social, economic and health impact has become better known and more widely understood. As acknowledged diseases of poverty, NTDs have risen up the global public health agenda and eliminating NTDs by 2030 is now a target within the Sustainable Development Goals (SDGs) [1]. Beyond the target itself, tackling NTDs is also critical to achieving Universal Health Coverage (UHC) and realising the Leave No-one Behind agenda, which have become two of the leading themes of the SDGs [2].

The World Health Organization (WHO) has developed clearly defined work plans for NTD elimination in Africa and Asia [3–5]. Through these, five intervention packages to prevent, control, treat and eliminate most NTDs have been identified:

- Preventive chemotherapy of population at risk/infected
- Intensified case-finding and management
- Integrated vector control/management
- Provision of safe water, sanitation and hygiene
- Veterinary public health.

Taking inspiration from the WHO NTD Roadmap [6], in 2012, donors, pharmaceutical companies, endemic countries and non-government organisations made the London Declaration commitment to end at least 10 NTDs by 2020. Consequently, progress has been encouraging in several areas, such as the systematic mapping of preventive chemotherapy and transmission control NTDs (PCT-NTDs) and the subsequent implementation of mass drug administration (MDAs).

However, as the NTD agenda has progressed, unsolved issues and new challenges have emerged, especially for PCT-NTDs, as have been highlighted by several NTD experts [7–10]. This year marks the fifth anniversary of the London Declaration, offering a fitting opportunity to examine the gaps and challenges facing NTD control and elimination efforts, to think about how to maintain what we have already achieved, and develop new and innovative approaches for managing all NTDs.

This paper summarises some of the practical unmet needs, challenges and assumptions in the current PCT-NTD and (Innovative and) Intensified -Disease Management NTD (IDM-NTD) programmes. Concrete steps for moving existing interventions towards more sustainable and integrated NTD approaches are laid out, as well as suggestions on operational aspects that should be prioritized in future NTD interventions.

## (Main text)

### Unmet needs, new challenges, and unspoken assumptions

In the last few years, debates at international conferences [11–14] have been providing forums for a broad range of NTD stakeholders to discuss urgent unmet needs and explore new approaches to tackle emerging challenges related to the elimination and post-elimination phase. Debates have been focusing on MDA and post-MDA phase, mapping and monitoring disease transmission, community engagement, and disease morbidity management. Furthermore, whilst in the past the debate has focused mainly on PCT-NTDs, the new global drive to eliminate all NTDs requires a consideration of how best to integrate IDM-NTD within current NTD programmes and health structures, introducing new elements in the discussion such as how to roll out an integrated NTD surveillance work plan and integrated vector management. In Table 1, some of the most relevant unmet needs and new practical challenges are summarised, based upon these discussions, the literature and field experience. The table does not provide an exhaustive summary of the current practical challenges in the NTD elimination roadmap (already partially discussed by others [7–10, 15]) but highlights some aspects of the key points in the debate.

**Table 1:** Summary of unmet needs, possible reasons for unmet needs, and new challenges and questions, grouped by main topics of debate

Main topics of the NTD debate	Unmet needs	Possible reasons for unmet need	New challenges and questions
Mass Drug Administration (MDA) and post-MDA phase	Preventive chemotherapy and transmission control (PCT)-NTDs	PCT-NTDs	PCT-NTDs
	• Ensure access to MDA for adults where disease burden is high • Ensure access to MDAs for disable individuals • Ensure treatment at PHC level for those that may have missed MDAs	• Limited financial support to target adults in MDAs • No drug donation available for adults during MDAs • Drugs donated for MDAs are not available at the PHC level outside campaigns • Possibly not adequate attention to disables and their need to access to treatments	• Can drug donation be extended to adults (where needed) and be made available at the PHC level? • Low prevalence reached in some areas where MDA has been ongoing for several years – is MDA still cost-effective if infection is very low (5%–10% prevalence)? • Risk of disease re-occurrence and/or increase back to initial levels if MDAs are stopped after reaching very low disease prevalence • MoH very likely in need to take on cost of treatment (including adults) in post-MDA, and/or post elimination phases if there is disease recurrence, and affected individuals still present
	Intense Disease Management (IDM)-NTDs	IDM-NTDs	IDM-NTDs (& non NTDs)
	• Roll out of blanket treatment for some IDM –NTDs easily managed with annual routine treatment (e.g. yaws)	• Disease mapping not performed as sensitives and specific RDTs not currently available (hence routine treatment cannot be rolled out)	Other diseases that were controlled by MDAs (e.g. scabies, strongyloidiasis, teniosis, cysticocercosis) could become a challenge after MDA campaigns stop, and health structures do not have adequate resources to treat these diseases
Re-mapping after MDA cycle Monitoring disease transmission	PCT-NTDs	PCT-NTDs	PCT-NTDs
	• Mapping of hypo endemic areas after an MDA cycle • Monitoring disease transmission in pre-elimination stage for certain PCT-NTDs	• Limited availability of sensitive and specific rapid diagnostic tests and /or laboratory tests to assess current infection and disease transmission in low endemic areas	• How would it be possible to confirm disease elimination, and absence of disease recurrence in post-elimination phase if disease transmission and/ or incidence cannot measured?
	IDM-NTDs	IDM-NTDs	IDM-NTDs
	• Disease burden for many IDM-NTDs and in many countries, where historically reported (Mapping of these diseases not performed)	• Limited tools and RDTs to map these diseases • Limited knowledge on how to triage, diagnose, confirm and report cases at the PHC level, for information on disease burden • Limited resources in laboratories at the heath care level to confirm disease transmission	• How would it be possible to confirm disease elimination, and absence of disease recurrence if the real burden of some of these is not yet known?
Community and community health workers engagement (CHW = personnel either working on a volunteer basis or occasionally compensated with incentives)	PCT-NTDs	PCT-NTDs	PCT-NTDs
	• Engagement of the community in public health interventions to take into account their specific needs • Community technical support, supervision and motivation for the sustainability of ongoing community health interventions • Financial recognition of the work already performed by CHWs in MDAs and in integrated MDAs	• Top-down approaches preferred in public health interventions • Ministry of health limited financial and human resources to support communities in disease prevention, treatment and management (when it could be done at community level) • Local/national financial limitations for the formalization of CHWs’ role in the health structure, and absence of a clear plan on how to do it	• How can the last cases be found in an elimination context if communities are not educated and sensitised? • Are the governments willing to retain CHWs and ensure the sustainability of their engagement?
	IDM-NTDs	IDM-NTDs	IDM-NTDs
	As above	As above	• CHWs workload likely to increase in interventions aimed at disease detection and management at the community level
Primary health care structure: Case finding and confirmation Surveillance Recording disease incidence	PCT-NTDs	PCT-NTDs	PCT-NTDs
	• Availability of clear case definitions for suspected and confirmed NTD cases at the health structure level • Guidance on case finding (active finding) • Adequate training on patient triaging procedures • Adequate laboratory resources to confirm NTD cases • Guidance on how to report disease incidence • Development of operational NTD surveillance strategies, and work plans • Revised reporting templates reflecting countries NTD reporting priorities	• Absence of clear guidance at the national level, on disease surveillance approaches • Very limited monitoring and evaluation process to assess data quality in health data routine reports provided by health facilities • Iinternational guidelines on surveillance not in line with the new vision on NTD elimination or simply non existent • Health information system reporting templates not adequate to report NTDs • Inadequate health data reporting process from decentralized health structures to central level • Limited national and international interest and \financial support in disease surveillance and in setting disease surveillance strategies	• Post MDA surveillance – how can it be set up if the health system in place does not have enough technical, human and financial resources to implement disease surveillance? • Emerging and re-occurring diseases: how would it be possible to ensure that diseases that have been eliminated, do not reappear due to neighbouring endemic countries, migrations, wars, and political instability?
	IDM-NTDs	IDM-NTDs	IDM-NTDs
	As above	As above	• Inability to obtain disease incidence and disease trends • Low likelihood to confirm disease elimination if disease transmission or incidence are not obtainable
Disability prevention Intense case management Home care management	PCT-NTDs	PCT-NTDs	PCT-NTDs
	• Disability and complications related to untreated PCT-NTDs and to IDM-NTD adequately addressed in the NTD elimination plans • Resources to ensure home care management for morbidity related to untreated NTDs	• Disability management related to NTDs not a public health priority in NTD national plans developed in the last decade • Limited financial support for disability prevention and case management	• People impacted by severe complications related to untreated NTDs are likely to be those individuals living in remote areas – how do we reach them and how can we ensure access to adequate health?
	IDM-NTDs	IDM-NTDs	IDM-NTDs
	• Adequate resources for managing patients with these diseases at the PHC level • IDM-NTD case management addressed in the NTD elimination plans	• These diseases have not been adequately addressed in NTD national plans developed in the last decade • Limited interest in managing these disease due to difficult management, and inadequate resources for disease detection	As above
Integrated disease prevention, management and surveillance of all NTDs	PCT & IDM-NTDs	PCT & IDM-NTDs	PCT & IDM-NTDs
	• Integration of these diseases not addressed so far as NTD programmes were generally disease focused (vertical approach), or PCT focused (with integration limited to PCT NTDs)	• Disease focused approaches generally preferred by donors (as easy to assess in terms of outcomes and impact), and more manageable at the MoH level • MoH NTD master plans generally referring to PCT NTDs only as priority, and not adequately addressing IDM diseases, more difficult to manage	• Integration of disease management at the MoH level will be challenging due to human resources and programme structure specifically establish for disease focused programmes • Fear of losing positions, jobs and power at every level of the health structure if disease management integration is performed • Donors to be convinced for allowing financial support initially provided for one disease, to be used for several diseases that may be more effectively dealt with if integrated
Integrated Vector Management	Vector borne NTDs (PCT and IDM)	Vector borne NTDs (PCT and IDM)	Vector borne NTDs (PCT and IDM)
	• Testing current vector control strategies in terms of entomological and epidemiological efficacy to assess what works and what does not work • Integration of entomological surveillance and vector control strategies in the national NTD prevention plan • Development of new insecticides to cope with emerging insecticide resistance	• Absence of adequate financial support for the development of new insecticides • Limited technical resources on integrated vector control • Limited guidance on how to assess what is actually feasible and cost effective • Limited human resources in developing and piloting new integrated vector control strategies • Integrated vector control has not been a priority so far in endemic countries • Vector control for NTDs has not been fully exploited as opportunity for disease prevention • Lack of international interest in vector control for vector borne NTDs	• Impact of climate change on vector distribution: how do we challenge the spreading of vectors and the subsequent emerging and re- occurring of vector borne diseases if vector control has been so far an untapped opportunity for disease prevention? • Undeveloped integrated vector control framework in some low income countries, especially in the African continent: how to we address this gap considering the current emerging vector borne diseases?

Specifically, as previously highlighted by others [9, 10], and in Table 1, MDA presents several challenges for achieving NTD elimination. Whether countries decide to stop MDA because a disease burden has fallen significantly, or increase it to achieve elimination of a particular disease, the challenges would be very similar. This is because neither low burden nor eliminated diseases are likely to remain a public health priority, meaning that international financial support may stop and domestic financing may be redirected towards more urgent issues. In both cases, diseases could potentially bounce back to previous levels of prevalence.

Current debates have also highlighted the need to understand better how to engage the community in NTD interventions. Research has shown that community engagement, education and sensitisation are highly important for the effectiveness of public health interventions [16–18], as well the importance of initiating community based interventions, particularly for NTDs [19].

As an essential component of community engagement, community health workers (CHWs) play a key role in many NTD interventions [20]. However, as their workload has increased, the absence of corresponding and adequate technical and financial support has generated concerns about how to retain CHWs and sustain the quality of their services. The need for integrating these individuals into the primary health care (PHC) system has already been highlighted, specifically in onchocerciasis programmes [21]. Nevertheless, the role of CHWs in delivering health services has not been addressed adequately [22, 23], creating further obstacles to NTD elimination efforts.

Recent publications [24, 25] have also highlighted the urgent need to develop new rapid diagnostic tests (RDTs), drugs and insecticides, driven partly by the demand from NTD-endemic countries where these commodities would be deployed. However, for these commodities to be used routinely and effectively in a health care system, it would require:

- The presence of a health system with sufficient human resources, technical skills and adequate staff competencies to be able to engage immediately in the introduction of these commodities
- The acceptance of new approaches to diagnose and manage NTD patients
- The presence of a solid monitoring and evaluation framework to monitor the use of these new tools
- A functional disease surveillance process in place to provide routine data about disease burden and disease trends.

As Table 1 highlights, these requirements do not reflect the current situation in many low income countries, where there are often significant gaps in capacity and technical knowledge at the health care delivery level [15]. The cost of using RDTs routinely in a primary health care setting may also be prohibitive, which could lead to governments using them solely as active surveillance tools to limit the overall cost. Such targeted use, however, does assume that there is some sort of surveillance strategy already in place for certain NTDs, which is very unlikely in many settings. Prioritising surveillance has been advocated by others in the sector [26–31], however very little has been done to ensure that this component of NTD management is included in NTD interventions.

Most of the current practical obstacles and unmet needs would not be completely resolved by the development and deployment of new commodities. An equally urgent priority is strengthening the capacity of the health care system and integrating NTD diagnosis, treatment and management so that communities can access health services via CHWs at the primary healthcare level. Routine disease surveillance should be established and integrated throughout the health system. Operational research is needed to understand how best to achieve this. Ideally it should be conducted before, or at least at the same time as, the introduction of new products.

### Looking forward: operational steps and priority areas for NTD elimination and beyond

**Fig. 1:**
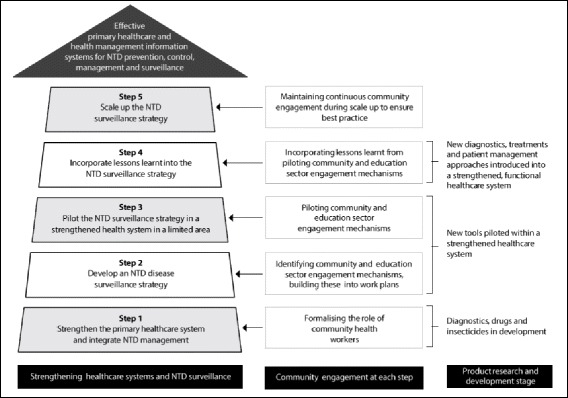
Priority steps to be taken in the path towards NTD elimination and post-elimination. In this figure, three main areas are highlighted: health care system, community engagement and research and development, and how the community engagement and the research and development areas interact with the main priority (strengthening of the health care system)

Figure 1 outlines five concrete steps to build sustainable NTD capacity in developing countries. Although these steps can overlap and be carried out concurrently, they have a logical flow and the risk of failing to follow them risks the mistakes of the past being repeated. If that happens, we are likely to find ourselves in the exact same situation in several years’ time – with similar challenges and unmet needs, the re-emergence of NTDs that had been eliminated and a health system unable to cope. Participation of the community remains crucial to this process as explained below.

### The role of CHWs, community and education sector

Community and CHW engagement is essential for the success of any PHC intervention. Chosen from within the community, CHWs are often the main point of contact with the formal health system for remote communities where health facilities are not easily accessible. They can play a fundamental role in disease prevention, control, management and surveillance.

To resolve the ongoing challenge of CHW retention, Ministries of Health could consider the option of offering fully-paid health professional roles to CHWs, or other paths to development. Such strategies have already been implemented by some African countries, through the introduction of monthly salaries or other performance based mechanisms [20, 32–34]. The approach has also been suggested by WHO [22] for other health programmes that engage with CHWs. There is no single approach to formalising the role of CHWs, however it is important for them to receive routine training, supportive supervision and constructive feedback. This could help ensure CHWs’ satisfaction and retention in the long run, as well as build capacity in the health system. While the integration of disease management and strengthening of the health system is ongoing (Step 1), the formalisation of the role of CHWs should be a parallel activity.

Community and education sector engagement should begin during Step 2 (see Fig. 1) and continue throughout the process of building NTD capacity in the health system. The education sector, especially primary schools, play a central role in the life of rural communities and can therefore provide a useful connection to vulnerable groups, primarily children and mothers. Schools can provide a public space where community awareness about NTD prevention and control can be increased and disease-related disability and associated stigma discussed, understood and accepted. Engagement of this sector can reinforce community engagement by ensuring that messages delivered are consistent and reiterated in schools and other community contexts. Schools could be involved in many public health interventions targeting NTDs, such as routine MDAs, educational projects focusing on waste management and water storage for prevention of vector borne diseases, and school activities that teach hygiene and sanitation to help prevent worm infections and other diseases related to poor hygiene practices.

During Step 2, when the NTD surveillance strategy is being drafted, mechanisms should be built in to encourage meaningful and effective community engagement, making use of CHW networks. These mechanisms for engagement should then be tested and refined as part of Step 3.

#### Step 1: Integrating NTD management and strengthening the primary healthcare system

The first step is to shift from vertical approaches, in which one disease is tackled at a time, to a more horizontal system where disease management (including all aspects of management, e.g. prevention, diagnosis, treatment and surveillance) is integrated - not only for different NTDs but also with other communicable and non-communicable diseases.

So far, integration has been applied for MDAs (e.g. when several drugs for treating different NTDs are delivered at once) and to some extent, in disease mapping (e.g. schistosomiasis and soil-transmitted helminth infections). The need now is to adopt an integrated approach at the PHC level. CHWs could be the connection between the community and the health care facility, and support the health staff in the routine implementation of these integrated preventive interventions. Health facilities could have a pivotal role in supporting the community in integrated vector surveillance and control [35] to prevent several NTDs that are carried by the same vector (e.g. *Aedes aegypti* control for dengue and chikungunya), or different vectors that have a similar behavioural pattern and can be controlled with a single strategy [36, 37].

The integration of disease control and management at the PHC level could be implemented for all diseases that have similar symptoms, such as NTDs that cause skin conditions. This approach would involve developing user-friendly triage tools that enable health staff at the PHC level to recognise diseases, and differentiate between them. Several publications have already highlighted examples of how the diagnosis and treatment of both NTDs and non-NTD skin-related diseases can be combined, and the benefits of doing so [38, 39]. It should be noted that grouping diseases that have very similar early signs, symptoms and morphology would not indicate a return to more vertically-oriented programmes. Rather, it would help to define more cost-effective approaches to managing NTDs aimed at supporting the PHC staff in their routine diagnosis of diseases that are hard to differentiate between. Taking into consideration existing levels of knowledge and limited resources, clinical (and pictographic) algorithms could be developed to start the process of addressing case detection, differential diagnosis and case referral for confirming suspected cases. Once these tools and processes are validated, training of health staff, especially at the PHC level, and the adequate provision of laboratory equipment, essential medicines and medical supplies for basic patient management should follow.

The benefits of strengthening the health system, especially at the first point of access, are for instance:

- Improved capacity for delivering routine disease prevention interventions at the community level (with the support of CHWs)
- Improved capacity for health facilities to take responsibility for routine NTD diagnosis and treatment through improved health worker knowledge, increased laboratory skills, and the provision of laboratory equipment and medicines
- An increased capacity for early NTD case detection, enabling health facilities to manage diseases more effectively, including cases that might recur even after elimination is achieved
- Improved reporting of NTD cases to the central level and quality of health data coming from decentralised health care facilities

During this step, it is important that the role of CHWs is formalised and their essential contribution to successful implementation is recognised, and as such, compensated. CHWs could have an important role in the following activities:

- Encouraging community attendance during health campaigns (e.g. vaccinations and MDAs)
- Advocating for community-based morbidity management and acceptance of disabled individuals (e.g. those affected by elephantiasis and hydrocele)
- Supporting activities associated with disease prevention (e.g. integrated vector surveillance and control approaches, such as reducing vector breeding sites for dengue prevention or implementing specific hygiene practices to prevent worm infections)

As part of efforts to integrate NTDs with routine disease management activities at the health facility level, gaps in the health management information system (HMIS) reporting process would be highlighted during this first step. Challenges could include the absence of HMIS indicators for specific NTDs, or the use of indicators that are unable to distinguish between suspected and confirmed cases. These would be noticed at this step, and addressed to achieve a system in which a comprehensive set of reliable and relevant NTD indicators are included, and fully integrated into the HMIS reporting tools in use for other conditions and diseases. During this first step, NTD reliable data would be collected in order to provide the baseline NTD epidemiological information necessary to understand disease incidence. This would help to define epidemiological thresholds and identify areas where more resources should be invested in order to strengthen active disease monitoring and prevent outbreaks.

#### Step 2: Developing an NTD surveillance strategy

Once capacity has been built to correctly detect and confirm diseases, adequately manage and treat patients and routinely capture NTD incidence via HMIS reporting, the next step is to develop a disease surveillance strategy. Based on routinely collected data and country priorities in selecting which disease to report and notify, a surveillance strategy could be drafted. For instance, in areas bordering countries that are highly endemic for one specific NTD, sentinel sites to observe potential changes of disease trends could be set up. Likewise, in areas where a specific disease has not been reported, but the population is still exposed to environmental risks, sentinel sites may be needed as warning system for disease re-emergence.

The surveillance strategy should take into consideration available resources and national public health priorities. Eventually, the goal of this strategy would be to provide an efficient mechanism to monitor disease trends. The strategy should also contain practical approaches and processes to ensure that data collected at the community level is delivered to the central office for analysis in real time, providing governments with an accurate estimation of changing disease burdens with which to identify potential outbreaks and plan public health responses. This would also enable each sub-district unit to define their own surveillance strategy for all relevant diseases.

NTD surveillance is crucial for disease control, elimination and post-elimination preparedness, and should therefore be the starting point for future NTD public health interventions. Starting with the already available diagnostic tools, drugs and expertise, it would be possible to improve the process of disease detection, confirmation, treatment and management.

During this step, CHWs also have an essential role to play, such as in supporting the mapping of diseases of interest (especially intense case management diseases such as Buruli ulcer) and establishing a community-based surveillance system for early detection and reporting of suspected cases to primary health facilities (e.g. lymphatic filariasis detection [40]).

#### Step 3: Piloting the NTD surveillance strategy

Implementing an effective NTD surveillance system with concrete community engagement is not an easy task. There are various models and approaches, while local contexts require an additional layer of adaptation. Therefore it is important to pilot different models in defined areas of the country to assess their feasibility. In this phase, it is essential to outline a clear monitoring and evaluation (M&E) framework to record input, process, output, outcome and impact indicators, and to ensure a quality assurance process throughout implementation. It is also highly advisable to establish an independent monitoring process to ensure data quality and reliability.

During this step, new drugs, RDTs and insecticides could be trialled and tested for effectiveness within a structure that has already been strengthened. Where the community is already engaged, this can also provide a clear indication of the viability of the new tools in the local context. The piloting process can take some time and the cost effectiveness of this step will have to be carefully assessed and incorporated into sustainability calculations with regards to the value for money NTD elimination provides.

CHWs would once again play an important role during step three, by supporting the piloting of proposed approaches, and, if needed, developing innovative, context-specific community-based solutions to combat NTD burdens.

#### Step 4: Lessons learnt

The lessons learnt from piloting each approach, including what worked, what did not and what could be improved upon, needs to be recorded and analysed. This analysis must include a cost evaluation for each model of surveillance. An important part of this process is sharing learning from localised pilot studies in different areas of a country so that approaches that fit a wide range of contexts can be developed, and a broad base of best practice evidence created.

Lessons learnt must be incorporated into the final NTD surveillance strategy prior to scale up, including any improvements that need to be made to the community and education sector engagement mechanisms. It is also paramount that the M&E component, including routine supervision and performance assessment, as well as routine assessment of collected health data, is part of the final strategy.

#### Step 5: Scale up

The fifth and final step is to scale up the finalised NTD surveillance strategy to the national level. This will have financial, operational and political implications. By following the previous steps, there should be strong evidence demonstrating the effectiveness and value for money of the approaches, tools and processes included in the NTD surveillance strategy, which should provide the justification for the adoption of necessary national policies.

### Further considerations

#### International financial support

The steps outlined above require some shift in terms of investment priorities. If financial investment has so far been dedicated to activities that are mostly single disease oriented, future investments should prioritise a set of interventions aimed at integrating NTD management within the PHC system, at least in those areas where the disease burden has already decreased and vertical programmes targeting specific diseases may no longer be cost effective. A shift towards an integrated approach, fully endorsed by donors, would also facilitate integration of diseases at the national level, especially where financially supported vertical health programmes may pose a challenge for Ministry of Health to operationalize this shift.

In the medium to long-term, funding from international donors will be necessary to build capacity and support the scaling up of effective strategies. There should then be a structured transition over time of financing responsibility from donors to national government, so that the services and capacity which have been painstakingly built are not lost due to budgetary constraints. This is particularly important when it comes to preserving mechanisms for community and education sector engagement, which are fundamental to the sustainability of any intervention.

#### Monitoring and evaluation framework

This shift towards integration requires a greater emphasis on M&E which, despite being advocated for in the past [26], remains one of the main neglected aspects of the NTD agenda. Robust M&E, with strong quality assurance mechanisms (including laboratory quality assessments) and the adequate supervision of processes to ensure collection of reliable health data, is a critical component throughout the five steps outlined above. If the impact of NTD interventions are to be measured and their value for money successfully assessed, then an effective M&E framework is essential.

Roughly 5–10% of an intervention budget should be allocated to M&E to measure the impact of that intervention [41] and inform the lessons learnt. An M&E framework should include the routine recording of process, output, outcome and impact indicators, such as the number of detected and confirmed NTD cases, the number of health facilities where disease trends have triggered active epidemiological investigations, and the number of outbreaks detected early enough to trigger preparedness measures. It is important to note that a strengthened health system and an effective surveillance strategy may lead initially to an increase in the recorded disease incidence.

International stakeholders involved in supporting NTD activities in endemic countries, including NGOs and private sector contractors, should ensure routine assessment of the quality of the field work by adequately budgeting for data quality assessments and ensuring an independent monitoring of these activities. They should also support Ministries of Health to establish sustainable systems for routine PHC staff supervision and feedback in order to improve all aspects of patient management, and for health data collection for surveillance. Internal capacity in organisations supporting NTD activities should be adequate to provide effective technical and financial support for all M&E activities.

#### Drug donation

An open discussion is needed about the possibility of introducing drug donations at the PHC level. Although medicines for individual treatment and care for IDM diseases have already been made available to health facilities, drugs for PCT- NTDs are currently donated for use in MDAs but not for routine PCT-NTD treatment. As part of current donation agreements, drugs left over from completed MDAs cannot be used at health facilities to treat patients, but must instead be kept for future campaigns. However, if financial support for subsequent MDAs is not available, treatments may expire and be wasted. This could be resolved with a routine drug distribution system to PHC centres based upon the number of cases reported.

#### Access to technical support

There are many excellent international guidelines for NTDs that are publicly available, however some, such those on surveillance [42], need to be updated to include more recent NTD case definitions. Furthermore, the highly practical and user-friendly international guidelines on disease prevention, control and management [37, 43, 44] should be amended to include NTD reporting, if needed and as per country priorities, and made more accessible to PHC personnel, many of whom are in need of more technical support and guidance. Based on field observation, knowledge of the existence of these useful guidelines for health staff can be low, resulting in them being poorly adopted within health care structures especially in rural areas. More effective communication about the existence of these guidelines and facilitation in the access to these documents would help to ensure that these informative and useful guidelines reach the local level, where they are most needed.

#### Aligning with the SDGs

While above we have outlined the steps required to integrate NTDs into the health system and build a surveillance system, it is also important that the process is aligned with wider national and international efforts to strengthen health systems in pursuit of the SDGs [45]. Health system strengthening is receiving increased interest from donors as part of the growing momentum behind achieving UHC and the health SDG (SDG 3) – in which NTDs are explicitly mentioned (SDG3.3) [46]. The central pillars of UHC are extending universal access to high-quality health services without causing financial hardship to users [47]. Clearly UHC cannot be achieved without a sustainable approach to eliminating NTDs that currently threaten 1.3 billion people and therefore they must be embedded within health system strengthening frameworks. Advocacy and engagement with donors and policy makers must focus on ensuring that the need to strengthen PHC for NTDs and NTD surveillance, and the supporting community engagement mechanisms, are included in this global agenda.

## Conclusion

This paper highlights some of the operational challenges that global NTD elimination efforts face, and the practical steps that could be taken to move this agenda forward in a sustainable way. The five suggested steps, and the crucial community and education sector engagement needed throughout the process, will require a strong commitment from donors, national governments, NGOs and communities themselves. However, the long-term pay off will be strengthened NTD treatment and management services within the PHC system, hugely reduced NTD burdens and the capacity to respond to re-emerging and new threats alike. This is all possible with the technical knowledge, experience and tools that are already available, and can be achieved without creating parallel, unsustainable systems.

## Recommendations

- ➢ National governments could consider developing an **integrated NTD prevention, control, management and surveillance strategy**, using existing tools and knowledge. This could begin with **strengthening the primary health care system**, and initiating cross-talk and integration of vertically oriented programmes ongoing in the country. Disease-oriented donors could consider supporting this shift towards more integrated approaches for country benefit and programme sustainability.
- ➢ National governments could strengthen **community engagement** through the **formalisation of community health workers**, ensuring that they are suitably supervised, trained, motivated and incentivised. The education sector could be engaged as a powerful link to the community.
- ➢ 5%-10% of the budget for NTD interventions could be allocated to setting up an **M&E framework** in order to develop a better understanding of what works, what is more cost-effective and whether the intervention represents value for money. Within this framework, donors, NGOs and ministries of health would all have an essential role in ensuring provision of effective supervision, technical support, and quality assurance mechanisms to obtain reliable data at the primary health care level.
- ➢ Pharmaceutical companies could consider **donating drugs for routine NTD treatment** and management at the primary health care level, and **extending donation to treatment of adults**, once capacity in confirming cases is built.
- ➢ International health organizations and NTD experts could advice on how to **improve the current health information management system** reporting templates **and international NTD surveillance guidelines** in order to reflect current NTD reporting needs and elimination goals. Access and adherence of country health staff to already available and up-to-date guidelines for NTD prevention, control, management and surveillance could be improved.
- ➢ Donors and national governments could agree a funding timetable, so that the **responsibility for financing NTD** prevention, treatment, management and surveillance is **transitioned to national governments** in a structured and sustainable manner.

## Additional file

Additional file 1: Multilingual abstracts in the five official working languages of the United Nations. (PDF 559 kb)

## Abbreviations

CHW: community health workers
COR-NTD: Coalition for Operational Research on NTDs
HIMS: health information management system
IDM: intense disease management
M&E: monitoring and evaluation
MDA: mass drug administration
NGDO: non-governmental development organizations
NTD: neglected tropical diseases
PCT: preventive chemotherapy and transmission control
PHC: primary health care
RDTs: rapid diagnostic tests
SDG: Sustainable Development Goals
UHC: Universal Health Coverage
WHO: World Health Organization
