# Supplementary material for: Neglected tropical diseases: exploring long term practical approaches to achieve sustainable disease elimination and beyond
Source: Infect Dis Poverty. 2017 Sep 27;6:147. doi: 10.1186/s40249-017-0361-8 (PMC5615470; doi:10.1186/s40249-017-0361-8)
Supplement: Additional file 1: — Multilingual abstracts in the five official working languages of the United Nations. (PDF 559 kb) [file 40249_2017_361_MOESM1_ESM.pdf]

Translation of the abstract into the five official working languages of the United Nations

الأمراض المدارية المهملة: استكشاف وسائل عملية طويلة الأجل لتحقيق معدل مُستدام من إزالة الأمراض وما بعدها.

جيوسيبينا أورتو، أوليفر ويليامز

ملخص

خلفية: تم إحراز تقدم ملحوظ في مكافحة الأمراض المدارية المهملة، ولكن تحديات جديدة قد ظهرت. الإبتكار في علم تشخيص الأمراض والعقاقير الأفضل والمبيدات الحشرية الجديدة، غالبا ما تُحدد كأولويات؛ غير أن الوصول إلى هذه الأدوات الجديدة قد لا يكون كافيا لتحقيق واستدامة القضاء على الأمراض، إذا لم يتم النظر في تحديات وأولويات مُعينة.

الهيكل الرئيسي: يلخص المؤلفون التحديات التشغيلية الرئيسية، وبناء عليها، يحددون أولويتين رئيسيتين: تعزيز قدرة النظام الصحي للرعاية الصحية الأولية في تشخيص الأمراض المدارية المهملة والتعامل معها بشكل صحيح؛ وإقامة تدابير فعالة لمراقبة الأمراض.

يُقترح اتخاذ خمس خطوات كإجراءات ملموسة لبناء خدمة رعاية صحية أولية فعالة للأمراض المدارية المهملة، ونظام معلومات للإدارة الصحية قادر على الإبلاغ بدقة عن هذه الأمراض. يُقترح إشراك المجتمع المحلي وإضفاء الطابع الرسمي على دور العاملين في مجال الصحة المجتمعية باعتبارهما عنصرين أساسيين في هذه الخطوات.

تحويل الدعم المالي من البرامج الموجهة للأمراض إلى إجراءات التدخل المتكاملة للمرض، وتحسين سبل الوصول إلى المبادئ التوجيهية الدولية لموظفي الرعاية الصحية الأولية، وتوافر الأدوية المتبرع بها في أنظمة الرعاية الصحية، هي أيضا من ضمن المعروضات كعناصر أساسية في العملية المقترحة.

الخلاصة: يستخلص المؤلفون أن الفشل في معالجة هذه الأولويات الآن قد يؤدي إلى مزيد من التحديات على الطريق الطويل نحو القضاء على الأمراض المدارية المهملة وما بعدها.

Translated from English version into Arabic by Mona Fahmy, through

TRANSLATORS
WITHOUT BORDERS

被忽视的热带病：探索实现可持续性疾病消除和额外预期的长期实用性方法

Giuseppina Ortu, Oliver Williams

摘要

引言：虽然在抗击被忽视的热带病中取得了显著进展，但仍面临新的挑战。研发创新性诊断方法、更好的药物和新型杀虫剂常被认为是当务之急。然而，如果不考虑某些关键问题和优先需求，仅仅获得这些工具并不足以实现和维持疾病的消除。

主要内容：作者总结了所面临的重大挑战，并基于此确定了两个主要的优先需求：加强初级卫生保健体系的准确诊断和管理被忽视热带病的能力，以及建立有效的疾病监测流程。

作者提出以 5 步法作为建立有效的初级卫生保健服务和能够准确报告这些疾病的健康管理信息系统的具体步骤。提出社区参与和规范社区卫生工作者角色是这些步骤的基本组成部分。

还提出将疾病导向计划的资金支持转移至疾病综合干预措施，提高初级保健人员对国际准则的可及性，以及为医疗保健机构提供捐赠药物，上述均是关键要素。

结论：作者认为不能解决这些优先需求将导致在被忽视的热带病的消除进程，以及日后漫长道路上面临更大挑战。

Translated from English version into Chinese by Peng Song, edited by Pin Yang

CHINESE CENTER FOR DISEASE CONTROL AND PREVENTION
NATIONAL INSTITUTE OF PARASITIC DISEASES

Les maladies tropicales négligées : exploration des approches pratiques pour parvenir à éliminer de façon durable les maladies et plus encore

Giuseppina Ortu, Oliver Williams

Résumé

Rappel des faits: Des progrès remarquables ont été accomplis dans la lutte contre les maladies tropicales négligées, mais de nouveaux défis sont apparus. Des diagnostics innovants, de meilleurs médicaments et de nouveaux insecticides sont souvent identifiés comme étant la priorité; toutefois, l'accès à ces nouveaux outils ne peut-être pas suffisant pour réussir et maintenir l'élimination des maladies si certains défis et les priorités ne sont pas pris en considération.

Partie principale: Les auteurs résument les principaux défis opérationnels et basé sur ceux-ci, identifient deux grandes priorités : renforcer la capacité du système de soins de santé primaires à diagnostiquer correctement et à gérer les maladies tropicales négligées ; et à établir un processus de surveillance efficace des maladies.

Cinq étapes sont proposés comme actions concrètes pour construire un service efficace de soins de santé primaire pour les maladies tropicales négligées ainsi qu'un système de gestion d'information de santé capable de rapporter avec précision ces maladies. L'engagement de la communauté et la formalisation du rôle des travailleurs de la santé de la communauté sont proposés comme des éléments essentiels de ces étapes.

Un changement du soutien financier d'un programmes axée sur les maladies vers des interventions intégrées des maladies, une amélioration de l'accès aux lignes directrices internationales pour le personnel de soins de santé primaire et une disponibilité de médicaments offerts dans les centres de soins de santé sont également proposés comme principaux éléments du processus proposé.

Conclusion: Les auteurs en viennent à la conclusion que l'incapacité de s'occuper maintenant de ces priorités pourrait mener à plus de défis dans ce long projet de l'élimination des maladies tropicales négligées et plus encore.

Translated from English version into French by AudreyRivard, through

TRANSLATORS
WITHOUT BORDERS

Забытые тропические болезни: изучение долгосрочных практических методов устойчивой ликвидации болезней

Джузеппина Орту (Giuseppina Ortu), Оливер Уильямс (Oliver Williams)

Аннотация

Предыстория: Значительный прогресс был достигнут в борьбе против забытых тропических болезней, но появились новые проблемы. Инновационная диагностика, более эффективные лекарств и новые инсектициды часто определяют в качестве приоритетных целей; однако доступ к этим новым инструментам может оказаться недостаточным для достижения устойчивой ликвидации болезней, если не учитывать некоторые проблемы и приоритеты.

Основной текст: Авторы обобщают основные оперативные проблемы и, исходя из них, определяют две основных приоритетных цели: укрепление потенциала системы здравоохранения, основанной на первичной медико-санитарной помощи, для надлежащей диагностики и лечения забытых тропических болезней, и создание эффективного процесса надзора за заболеваниями.

В качестве конкретных действий предлагается пять шагов по созданию эффективной службы первичной медико-санитарной помощи для лечения забытых тропических болезней и информационной системы службы здравоохранения для ведения точной отчетности об этих заболеваниях. Предполагается, что участие сообщества и формализация роли работников здравоохранения в общинах являются важными компонентами этих мер.

Также предлагается перенести финансовую поддержку с программ, ориентированных на болезни, на комплексные мероприятия по борьбе с заболеваниями, расширение доступа к международным рекомендациям для сотрудников учреждений первичного медико-санитарного обслуживания и доступность пожертвованных лекарственных средств в структурах служб здравоохранения как ключевые элементы предлагаемого процесса.

Заключение: Авторы делают вывод, что сегодняшнее игнорирование этих приоритетных проблем может в дальнейшем привести к проблемам на долгом пути к ликвидации забытых тропических болезней.

Translated from English version into Russian by Oksana Weiss, through

TRANSLATORS
WITHOUT BORDERS

Enfermedades tropicales desatendidas: Enfoques prácticos a largo plazo para una erradicación sostenible

Autores: Giuseppina Ortu, Oliver Williams

Resumen

Contexto: Pese a que se ha progresado mucho en la lucha contra las enfermedades tropicales desatendidas, también han surgido nuevos retos. Aunque generalmente se da prioridad a diagnósticos innovadores, medicamentos más eficaces y nuevos insecticidas, estos medios a menudo no bastan para erradicar la enfermedad y evitar su reaparición si no se tienen en cuenta determinados desafíos y prioridades.

Cuerpo principal: Los autores resumen los principales desafíos operacionales y, partiendo de estos, identifican dos grandes prioridades: reforzar la capacidad del sistema de atención primaria mediante el correcto diagnóstico y tratamiento de las enfermedades tropicales desatendidas, y establecer un proceso de monitorización de la enfermedad que resulte eficaz.

Para crear un servicio de atención primaria eficaz que dé respuesta a dichas enfermedades y un sistema de información y gestión capaz de reportarlas con exactitud, se proponen cinco pasos concretos. La participación de la comunidad y la formalización de la función del personal sanitario de la comunidad se proponen como componentes esenciales de estos pasos.

Otros factores clave del proceso propuesto son la transferencia del apoyo financiero de programas orientados a la enfermedad a intervenciones que la integren, un mejor acceso del personal de atención primaria a las pautas internacionales, y disponibilidad de medicamentos donados en las instalaciones médico-sanitarias.

Conclusión: Los autores concluyen que no abordar estas prioridades en la actualidad puede acarrear un mayor número de obstáculos en el largo camino hacia la erradicación de las enfermedades tropicales desatendidas.

Translated from English version into Spanish by Marta Callava Linares, through

TRANSLATORS
WITHOUT BORDERS
